# Early changes in ammonia levels and liver function in patients with advanced hepatocellular carcinoma treated by lenvatinib therapy

**DOI:** 10.1038/s41598-019-48045-z

**Published:** 2019-08-20

**Authors:** Kazuki Ohya, Tomokazu Kawaoka, Maiko Namba, Shinsuke Uchikawa, Kenichiro Kodama, Kei Morio, Takashi Nakahara, Eisuke Murakami, Akira Hiramatsu, Masataka Tsuge, Masami Yamauchi, Michio Imamura, Kazuaki Chayama, Hiroshi Aikata

**Affiliations:** Department of Gastroenterology and Metabolism, Hiroshima University Hospital, 1-2-3 Kasumi, Minamiku, Hiroshima, 734-8551 Japan

**Keywords:** Chemotherapy, Cancer metabolism

## Abstract

We aimed to investigate the early changes in ammonia levels and liver function in patients with advanced hepatocellular carcinoma treated with lenvatinib. This retrospective study included 23 patients with advanced hepatocellular carcinoma who were able to receive lenvatinib continuously for at least 1 week. We compared their ammonia levels (NH3), total bilirubin (Bil), albumin, and prothrombin (PT) activity at before and after 1 week of lenvatinib administration, and additionally, compared the 2 groups which were divided based on the presence/absence of portosystemic collaterals (PSCs). Before administration of lenvatinib the patients with PSCs had significantly worse ammonia levels and liver function than the patients without PSCs (NH_3_: *P* = 0.013, Bil: *P* = 0.004, PT: *P* = 0.047, respectively). Moreover, the indices were worse in all the patients after 1 week of lenvatinib than before administration (NH3: *P* = 0.001, Bil: *P* = 0.025, PT: *P* < 0.001, respectively). Moreover, the changes in ammonia levels were investigated for 4 weeks. The ammonia level increased, to peak at 2 weeks, but decreased after 3 weeks. None of the patients discontinued lenvatinib therapy because of an adverse event. The ammonia levels of the study patients increased from baseline at 1 week after lenvatinib administration, but therapy could be continued for 4 weeks by appropriate management.

## Introduction

In Japan, sorafenib, an oral multikinase inhibitor, has been used since 2009 for patients with advanced unresectable hepatocellular carcinoma (HCC) as first-line treatment^1,2^. No new drug options were available for patients with advanced HCC until 2017, when regorafenib became available as second-line treatment for patients with advanced HCC. In 2018, lenvatinib became available as a first-line treatment. Lenvatinib is also a multikinase inhibitor, and showed equivalent overall survival rates and a higher response rate than the response rate of sorafenib in the REFLECT study^3–5^. Now we can use sorafenib and lenvatinib as first-line and regorafenib as second-line for advanced HCC in Japan. Although there were few data about sequential therapy of these drugs, it is expected to improve the prognosis of patients with advanced HCC.

Sorafenib abrogates tumor growth by inhibiting tumor angiogenesis through inhibition of vascular endothelial growth factor receptors (VEGFRs) and platelet-derived growth factor receptors (PDGFRs) and affecting the proliferation/survival of tumor cells^6^. Sorafenib has been found to have unique side effects, such as hypertension and hand-foot-skin reaction (HFSR), which had not been reported for previous antineoplastic agents^7^.

The affinities of lenvatinib for VEGFRs and fibroblast growth factor receptors (FGFRs) are different from those of sorafenib^4,5^. Therefore the side effects of lenvatinib were expected to be different from those of sorafenib. There have been a few reports on hepatic encephalopathy associated with sorafenib treatment, which, like lenvatinib, is a multikinase inhibitor, but the reports are very sporadic^8^. Hsu *et al*. reported that sorafenib did not increase the risk of hepatic encephalopathy in cirrhotic rats, and Chiu *et al*. reported that the proportion of patients with advanced HCC and underlying Child-Pugh Class A cirrhosis who were treated with sorafenib and developed hepatic encephalopathy was 1.9%^9,10^. At present, there have been few reports available on the impact of lenvatinib on ammonia levels or the liver function of patients with advanced HCC treated in clinical practice, and the mechanisms of the antitumor activity of lenvatinib remain unclear. Therefore, in this study, we aimed to evaluate the impact of lenvatinib on patients immediately after its administration by retrospectively investigating the changes in ammonia levels and other liver function indices in patients with advanced HCC who were treated with lenvatinib.

## Patients and Methods

### Patients

We retrospectively reviewed the data from 23 patients who received lenvatinib therapy for advanced HCC at our institution between April and September 2018. All the patients underwent a radiological evaluation by contrast-enhanced computed tomography (CT) or contrast-enhanced magnetic resonance imaging (MRI), or underwent a needle biopsy, and were diagnosed with advanced unresectable HCC. At our institution, lenvatinib therapy is used for patients with advanced unresectable HCC with Child Pugh class A liver disease and an Eastern Cooperative Oncology Group (ECOG) performance status score of 0 or 1. We included patients in the study who satisfied the criteria for lenvatinib therapy and could take lenvatinib at least 1 week continuously without withdrawal or dose reduction.

### Lenvatinib administration

All patients received oral lenvatinib (12 mg/day for bodyweight ≥60 kg or 8 mg/day for bodyweight <60 kg). Dose reduction of lenvatinib was determined by the treating physician based on the criteria listed in the manufacturer’s package insert, as well as the extent of adverse effects (AEs). We evaluated laboratory data before, and at 1, 2, 3, and 4 weeks after initiation of treatment.

### Assessment of portosystemic collaterals

In a previous study we reported the risk factors for exacerbation of esophageal varices or portosystemic encephalopathy in patients with compensated cirrhosis^11^. We considered the following vessels to be portosystemic collaterals (PSCs): left gastric vein, posterior gastric vein, short gastric vein, paraesophageal vein, paraumbilical vein, and splenorenal shunt; and evaluated them on axial and coronal dynamic CT images. We measured the diameters of the PSCs with the largest portion of the vessels. The median values of these vessels were defined as cutoff diameters (6, 4, 2, 4, 3, and 13 mm, respectively). As in the previous report, we defined PSCs and the value of cutoff in this study except for splenorenal shunt. Moreover, splenorenal shunt and other PSCs over 10 mm were considered present.

### Ethical approval and informed consent

This study was carried out in accordance with the Declaration of Helsinki and all the applicable local regulations. The study protocol was approved by the Ethics Review Committee of the Hiroshima University. Informed consent was obtained from all individual participants included in the study.

### Statistical analysis

Numerical data were expressed as median (range). Differences between nonparametric variables in the patient groups were assessed by the Fisher exact probability test, Wilcoxon test, Kruskal-Wallis test, and Mann-Whitney U test. All two-tailed P values < 0.05 were considered significant. SPSS software (SPSS Inc, Chicago, IL) was used for all statistical analyses.

## Results

### Characteristics of patients

The clinical characteristics, results of laboratory testing, and tumor factors of 23 patients before treatment are summarized in Table 1. The median patient age was 74 (range 58–84) years, and 19 patients were men and 4 patients were women. Twenty-one patients had an ECOG performance status score of 0, and 11 patients had a Child-Pugh score of 5. Twelve patients received 12 mg of lenvatinib as the initial dose. Thirteen patients had been administrated any drugs for hyperammonemia such as branched-chain amino acid (BCAA), lactulose, carnitine and zinc before starting lenvatinib. It had been demonstrated that poor zinc status impairs nitrogen metabolism by reducing the activity of urea cycle enzymes in the liver and of glutamine synthetase in the muscle^12^. Patients with PSCs on imaging were judged as PSC-present. Consequently, 10 patients were considered PSC-present. Table 2 summarizes data on the PSCs. Three of 10 patients showed the following shunts: 24-mm superior mesenteric vein–testicular vein shunt, 10-mm splenic vein–internal iliac vein shunt, and 12-mm gastrorenal shunt.

**Table 1:** Clinical characteristics, baseline laboratory tests and tumor factors of study patients.

Factor	Total (n = 23)
Age (years)*	74 (58–84)
Gender (male/female)	19/4
Body weight (kg)*	59 (38–86)
Etiology (HBV/HCV/HBV + HCV/others)	3/9/1/10
Performance Status (0/1)	21/2
Child-Pugh Score (5/6)	11/12
Total bilirubin (mg/dL)*	0.8 (0.3–1.7)
Albumin (g/dL)*	3.6 (2.9–4.8)
Prothrombin activity (%)*	82 (69–100)
Aspartate aminotransferase (U/L)*	29 (17–73)
Alanine aminotransferase (U/L)*	18 (10–92)
Leukocyte count (/mm^3^)*	4530 (1770–10950)
Hemoglobin (g/dL)*	11.5 (7.9–16.8)
Platelet count (×10^4^/uL)*	13.3 (4.9–31.1)
Alpha-fetoprotein (ng/mL)*	162 (3.3–236900)
DCP (mAU/mL)*	1142 (16–287990)
Ammonia level (μmol/L)*	28 (10–79)
Intrahepatic tumor number (0/1/2–3/4≤)	4/3/0/16
Main tumor size (mm)*	21 (0–140)
Tumor size relative to the liver (<50%/50% ≤)	22/1
Macro vascular invasion (yes/no)	5/18
Extrahepatic metastasis (yes/no)	12/11
TACE refractory (yes/no)	11/12
HCC stage (II/III/IVa/IVb)	5/4/3/11
Initial dose of lenvatinib (8 mg/12 mg)	11/12
Portosystemic collaterals (present/absent)	10/13
Drug for hyperammonemia (BCAA/lactulose/carnitine/zinc)	13/4/1/1

*Median (range), DCP: Des-γ-carboxy prothrombin, TACE: transcatheter arterial chemoembolization, HCC: hepatocellular carcinoma.

**Table 2:** The number of each portosystemic collaterals of all patients.

Portosystemic collaterals	n (median [mm])
Left gastric vein	3 (5.4)
Anterior gastric vein	7 (6.5)
Short gastric vein	1 (6.5)
Paraesophageal vein	3 (8.0)
Splenorenal shunt	1 (7.9)
Superior mesenteric vein – testicular vein shunt	1 (24)
Splenic vein – internal iliac vein shunt	1 (10)
Gastrorenal shunt	1 (12)

The patients were divided into 2 groups based on the presence or absence of a PSC. Table 3 shows the clinical characteristics, results of laboratory testing, and tumor factors of the patients stratified according to presence or absence of PSCs. The differences between values for total bilirubin, prothrombin activity, alpha-fetoprotein, and ammonia between the 2 groups were significant.

**Table 3:** Comparison of characteristics, laboratory tests and tumor factors before treatment between patients with and without portosystemic collaterals (PSCs).

	PSCs (−) (n = 13)	PSCs (+) (n = 10)	p value
Age*	76 (64–84)	72.5 (58–83)	0.60
Gender (male/female)	10/3	9/1	0.41
Performance status (0/1)	12/1	9/1	0.85
Child-Pugh score (5/6)	8/5	3/7	0.13
Total bilirubin (mg/dL)*	0.6 (0.3–1.2)	0.85 (0.7–1.7)	0.004
Albumin (g/dL)*	3.6 (2.9–4.8)	3.5 (3–4.2)	1.0
Prothrombin activity (%)*	86 (73–100)	76 (69–97)	0.047
Aspartate aminotransferase (U/L)*	29 (17–73)	32 (23–73)	0.38
Alanine aminotransferase (U/L)*	19 (10–92)	16.5 (12–51)	0.82
Platelet count (×10^4^/uL)*	14.8 (7.5–31.1)	11.3 (4.9–15.7)	0.13
Alpha-fetoprotein (ng/mL)*	9.6 (3.3–3992)	1246 (24–236900)	0.012
DCP (mAU/mL)*	1142 (25–287990)	1153 (16–64229)	0.98
Ammonia level (µmol/L)*	25 (10–45)	50 (14–79)	0.013
Intrahepatic tumor number (0/1/2–3/4≤)	1/1/0/11	3/2/0/5	0.20
Main tumor size (mm)*	30 (0–60)	20 (0–140)	0.76
Tumor size relative to the liver (<50%/50% ≤)	12/1	10/0	0.37
Macro vascular invasion (yes/no)	2/11	3/7	0.40
Extrahepatic metastasis (yes/no)	6/7	6/4	0.51
TACE refractory (yes/no)	8/5	3/7	0.13
HCC stage (II/III/IVa/IVb)	3/3/2/5	2/1/1/6	0.74
Initial dose of lenvatinib (8 mg/12 mg)	7/6	4/6	0.51

*Median (range), PSCs: portosystemic collaterals, DCP: Des-γ-carboxy prothrombin, TACE: transcatheter arterial chemoembolization, HCC: hepatocellular carcinoma.

### Changes in indices of liver function between baseline and at 1 week after initiation of lenvatinib

**Figure 1:**
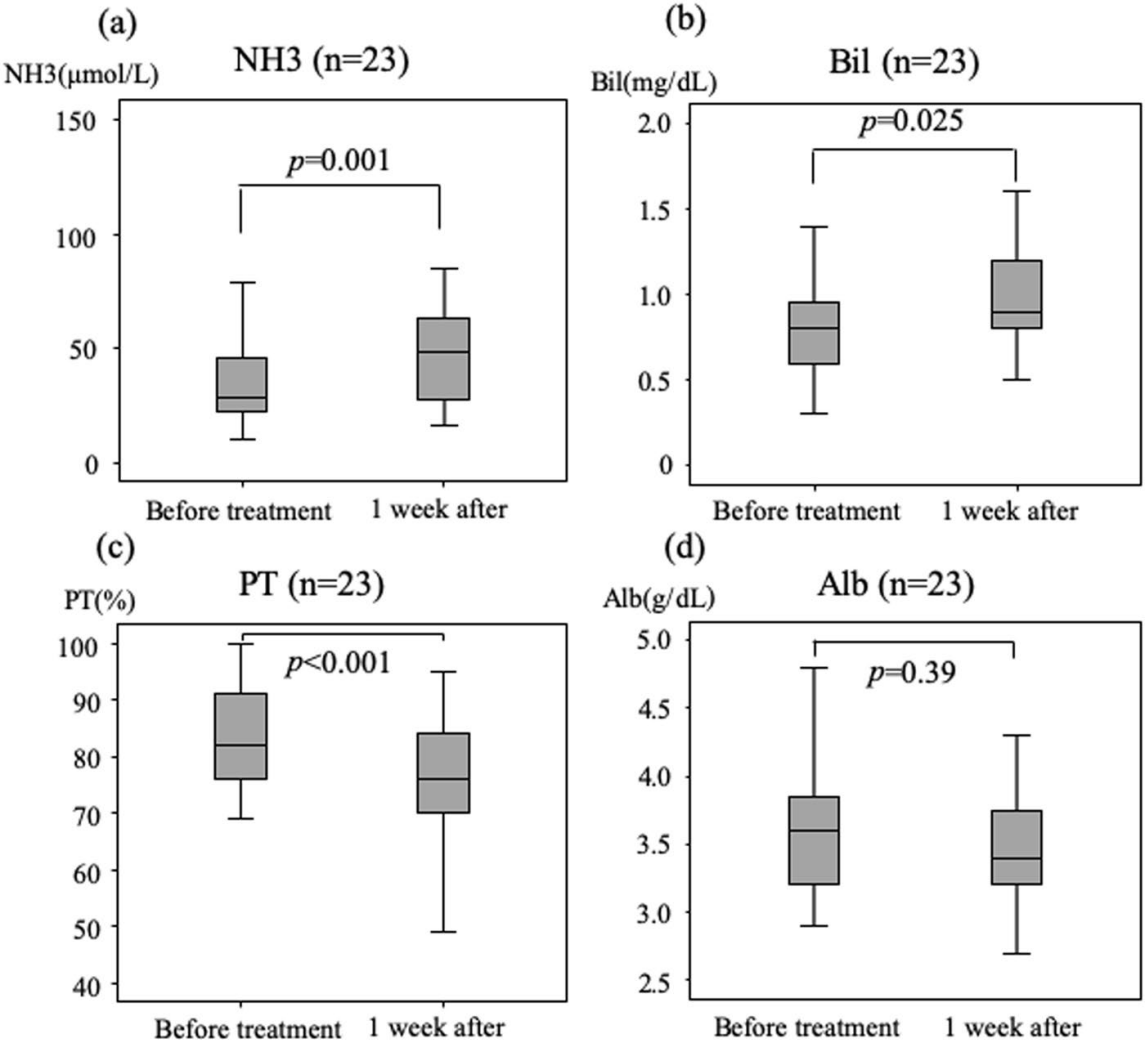
Changes in ammonia levels, total bilirubin, and prothrombin activity at baseline and 1 week after initiation of lenvatinib treatment. Differences between ammonia levels (**a**), total bilirubin (**b**) and prothrombin activity (**c**) at baseline and at 1 week after initiation were significant. Changes in albumin levels (**d**) were not significant.

Figure 1 shows levels at baseline versus at 1 week after start of treatment for all patients. The levels of total ammonia (median 28 vs 48 μmol/L, respectively; *P* = 0.001, Fig. 1a) and bilirubin (median 0.8 vs 0.9 mg/dL, respectively; *P* = 0.025, Fig. 1b) increased, and prothrombin activity (median 82 vs 76%, respectively; *P* < 0.001, Fig. 1c) decreased significantly. The difference between albumin levels (median 3.6 vs 3.4 g/dL, respectively; *P* = 0.39, Fig. 1d) was not significant.

**Figure 2:**
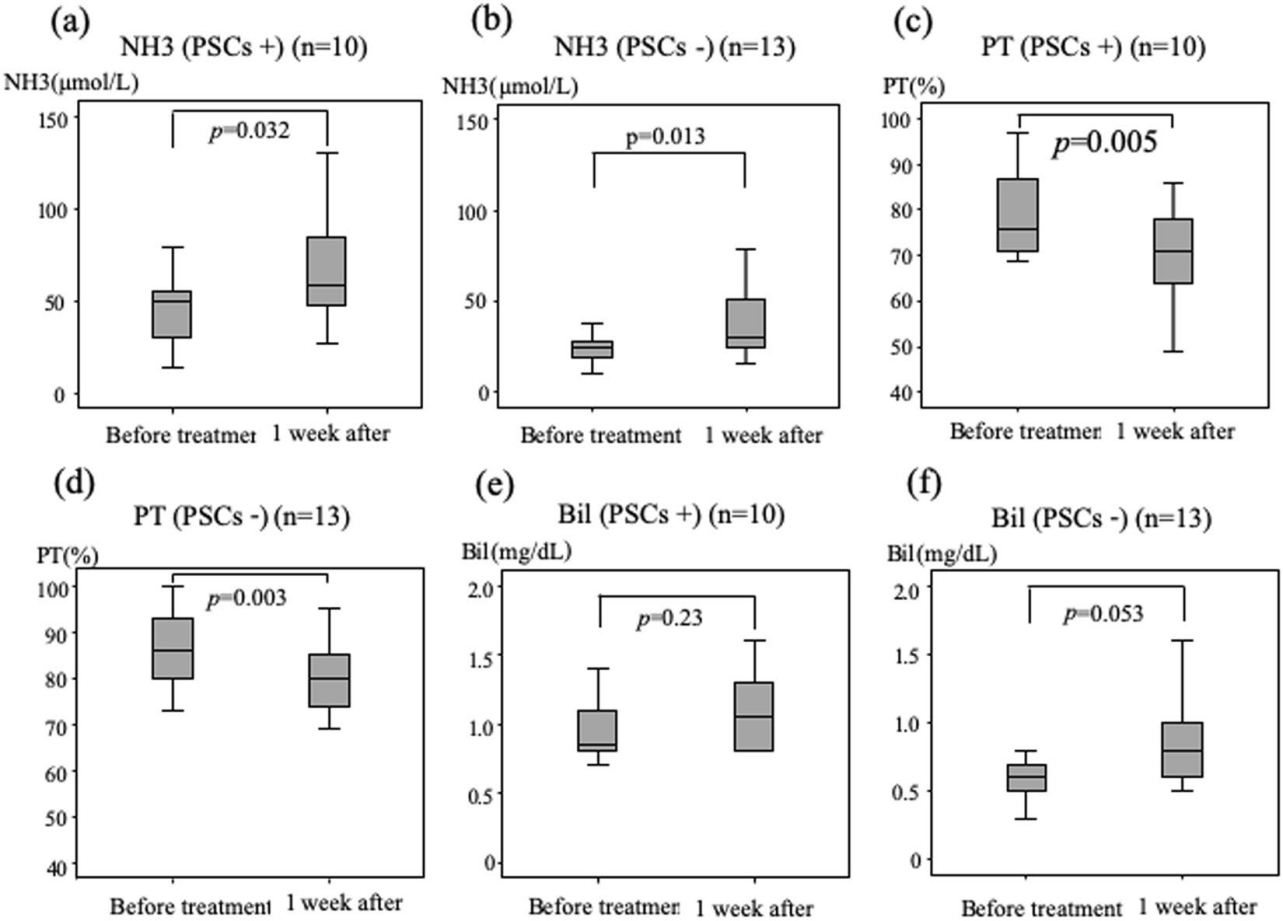
Changes in ammonia levels, prothrombin activity and total bilirubin at baseline and 1 week after initiation of lenvatinib treatment in patients without or with portosystemic collaterals (PSCs). Differences between ammonia levels and prothrombin activity at baseline and at 1 week after initiation of treatment were significant in patients both with (**a,c**) and without (**b,d**) PSCs. Changes in total bilirubin were not significant in either group (**e,f**).

Figure 2 shows levels at baseline versus at 1 week after start of treatment for patients with and without PSCs. The levels of total ammonia increased significantly in the PSCs-present (median 50 vs 59 μmol/L, respectively; *P* = 0.032, Fig. 2a) and PSCs-absent groups (median 25 vs 30 μmol/L, respectively; *P* = 0.013, Fig. 2b). The prothrombin activity decreased significantly in the PSCs-present (median 76 vs 71%, respectively; *P* = 0.005, Fig. 2c) and PSCs-absent groups (median 86 vs 80%, respectively; *P* = 0.003, Fig. 2d). However, the total bilirubin levels did not change significantly in both the PSCs-present (median 0.9 vs 1.1 mg/dL, respectively; *P* = 0.23, Fig. 2e) and PSCs-absent groups (median 0.6 vs 0.8 mg/dL, respectively; *P* = 0.053, Fig. 2f). The albumin levels also showed no significant difference in both the PSCs-present (median 3.5 vs 3.3 g/dL; respectively; *P* = 0.24) and PSCs-absent groups (median 3.6 vs 3.7 g/dL, respectively; *P* = 0.88).

On the other hand, we compared the numbers of patients in the PSCs-present versus the PSCs-absent group whose ammonia levels were elevated greater than 80 µmol/L (Table 4). To our knowledge, the cut-off value of hyperammonemia had not been defined in general. However, Shalimar *et al*. were showed that an ammonia level of ≥79.5 µmol/L was associated with a higher frequency of organ failures^13^. So, we defined ammonia level of ≥80 µmol/L as hyperammonemia in this study. Three patients in the PSCs-present group had elevated ammonia levels greater than 80 µmol/L, whereas no patient in the PSCs-absent group had an elevated ammonia level greater than 80 µmol/L. The number of patients with high ammonia levels tended to increase in the PSCs-present group (*P* = 0.068).

**Table 4:** The number of patients whose ammonia level increased to greater than 80 µmol/L in patients with or without portosystemic collaterals.

	NH3 < 80 µmol	NH3 ≥ 80 µmol
Portosystemic collaterals (−)	13	0
Portosystemic collaterals (+)	7	3

Fisher exact test: *P* = 0.068.

### Safety of lenvatinib

Seven of 23 patients were able to continue lenvatinib at the same level as the initial dose, without any intervention. Sixteen of 23 patients underwent interventions up to 4 weeks after the start of lenvatinib, because of an AE. Interventions included the following: withdrawal or dose reduction of lenvatinib; medication for elevated ammonia level; and shunt occlusion, which was performed by interventional radiology. The following AEs occurred: elevation of ammonia level (6 patients), fatigue (5 patients), anorexia (4 patients), proteinuria, fever, liver function impairment (2 patients each), diarrhea, hand-foot skin reaction, and hepatic encephalopathy (1 patient). Until 4 weeks, various AEs were identified and needed management, but there was no patient who discontinued the therapy due to AEs.

### Changes in ammonia levels over 4 weeks of lenvatinib administration

**Figure 3:**
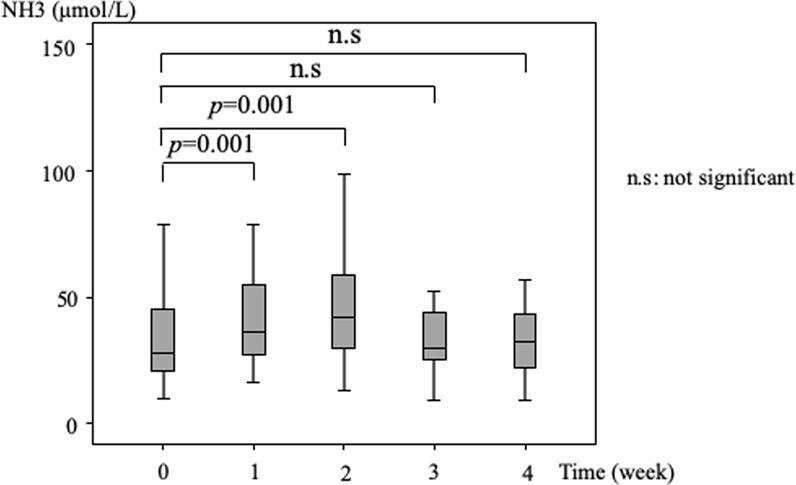
Changes in ammonia levels over a 4-week period. The ammonia level increased and peaked at 2 weeks after initiation of treatment. The differences between ammonia levels at baseline and 1 week after initiation of lenvatinib treatment and baseline and 2 weeks after initiation of treatment were significant. The ammonia levels then decreased, and the differences between baseline and 3 and 4 weeks were not significant.

Figure 3 shows changes in ammonia levels at baseline and at weekly intervals after the initiation of lenvatinib. The differences between baseline and 1 week and baseline and 2 weeks were significant (baseline vs 1 week: median 28 vs 48 μmol/L, *P* = 0.001; and baseline vs 2 weeks: median 28 vs 50 μmol/L, *P* = 0.001). At 3 and 4 weeks after administration, the ammonia levels tended to decrease, and the differences between baseline and 3 and 4 weeks were not significant.

**Figure 4:**
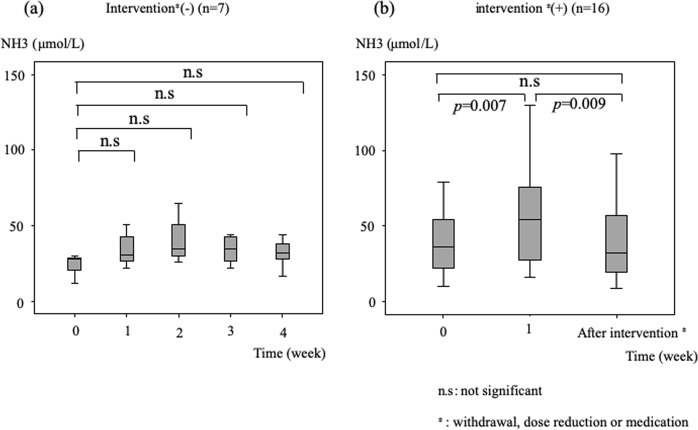
Changes in ammonia levels of patients not undergoing intervention and patients undergoing intervention. The differences between ammonia levels remained flat without significance between baseline and 1, 2, 3, or 4 weeks of treatment with lenvatinib in patients not undergoing intervention (**a**). The differences in ammonia levels between baseline and 1 week of treatment and between 1 week and after intervention were significant (**b**). The ammonia level decreased immediately after intervention.

Therefore, we divided the patients into 2 groups, based on whether or not they received some type of intervention. Figure 4 shows the changes in ammonia levels based on nonintervention vs intervention. Seven patients did not receive an intervention, and their ammonia levels did not show changes. The ammonia level remained flat without statistical significance between baseline and at 1, 2, 3, or 4 weeks (Fig. 4a). The intervention group consisted of 16 patients. Figure 4b shows the ammonia levels at baseline, at 1 week of administration, and at the first intervention. The difference between ammonia levels at baseline and 1 week was significant (median 37 vs 54 μmol/L, respectively; *P* = 0.007), and the difference between ammonia levels at 1 week and at the time of first intervention (median 54 vs 34 μmol/L, respectively; *P* = 0.009) was significant. There was only one patient who needed withdrawal or dose reduction and no patient discontinued the therapy due to hyperammonemia or hepatic encephalopathy in this study.

## Discussion

In this study, we investigated the early changes in ammonia levels and liver function tests after administration of lenvatinib to patients with advanced HCC. The ammonia level, total bilirubin, and prothrombin activity were significantly worse at 1 week than at baseline. Comparisons between PSCs-present and PSCs-absent patient groups showed that the PSCs-present group had a worse ammonia level, total bilirubin level, and prothrombin activity than the PSCs-absent group. Both groups showed significantly worse ammonia levels and prothrombin activity at 1 week than at baseline.

Changes in ammonia levels from baseline were investigated over a 4-week period. The ammonia level increased to peak at 2 weeks, but then started to decrease at 3 weeks. Among the patients who did not receive any intervention over a 4-week period from baseline, ammonia levels did not change significantly from baseline over that period. Among patients who received intervention, the differences between ammonia levels at baseline and 1 week and between 1 week and after the first intervention were significant. One patient developed hepatic encephalopathy and had to stop lenvatinib therapy. Since lenvatinib has just become available for clinical practice, its appropriate use requires further investigation.

We speculate that the significant changes in ammonia levels and liver function are due to decreased intrahepatic blood flow associated with lenvatinib. In previous studies, the authors concluded that intrahepatic blood flow is regulated by stellate cells that reside in the perisinusoidal space of Disse^14,15^. Several vasoconstrictor molecules such as endothelin and prostaglandin and the vasodilator molecule nitric oxide (NO) activate the stellate cells, and the contraction and relaxation of stellate cells regulate intrahepatic blood flow^14–17^. NO is produced by sinusoidal endothelial cells in response to various factors, such as endothelin and shear stress, and NO production is reduced in cirrhotic liver because of dysfunctional sinusoidal endothelial cells^18–20^. Mediators of angiogenesis, including VEGF, PDGF, and FGF, induce formation of the portal-systemic circulation^21,22^. VEGF also increases NO production via several mechanisms^21,23–26^. Lenvatinib inhibits this remaining salvage pathway, with subsequent further decrease in NO production. Therefore, the reduction in NO production induces contraction of the hepatic stellate cells, increases intrahepatic vascular resistance, and reduces intrahepatic blood flow. Hypertension is a frequent side effect of lenvatinib, and hypertension might be a result of reduced NO production, which induces an increase in systemic vascular resistance^3^. In other words, hypertension is a result of increase in systemic vascular resistance and hyperammonemia is a result of increase in intrahepatic vascular resistance, and both hypertension and hyperammonemia are effect of lenvatinib. Further study is needed to investigate changes in intrahepatic blood flow occurring with the administration of lenvatinib.

When patients were divided into 2 groups based on the presence or absence of PSCs, the differences between total bilirubin levels, prothrombin activity, and ammonia levels at baseline between the 2 groups were significant. This might be accounted for by the fact that patients with PSCs tend to have progressive liver cirrhosis and portal hypertension, which tends to lead to worsening liver function. Both groups showed significantly decreased prothrombin activity and increased ammonia level at 1 week of lenvatinib compared with baseline (total bilirubin and albumin did not show a significant difference in either group). These results suggest that in patients with PSCs in whom liver function had already decreased, their liver function might continue to decrease because of reduction in the intrahepatic blood flow associated with lenvatinib. We also found that a higher number of patients with PSCs had ammonia levels increasing to greater than 80 µmol/L than the number of patients without PSCs (Table 4). Therefore, we think that the risk of hyperammonemia might be higher in patients with than without PSCs. Thus, for patients with PSCs, shunt occlusion therapy should be considered before administration of lenvatinib. Additionally, treatment for hyperammonemia, such as lactulose or BCAA, should definitely be initiated.

The ammonia levels were checked over 4 weeks after initiation of lenvatinib. The levels increased significantly from baseline at 1 and 2 weeks after initiation of treatment. However, 3 weeks after administration, the ammonia level had decreased and the difference between baseline and 3 weeks was not significant. That finding was thought to be associated with the fact that most patients developed an adverse event related to lenvatinib and received an intervention, including withdrawal, dose reduction, medication, and shunt occlusion. Among the patients without intervention, the ammonia level did not increase significantly. While, among patients receiving an intervention, the ammonia level significantly decreased after intervention. In 16 patients receiving an intervention, 14 patients (88%) received intervention between at 1 and 3 weeks after administration. We speculate that this is the reason for decrease of ammonia level at 3 weeks after administration. Over the duration of this study, only 3 patients developed hyperammonemia, and one of 3 patients developed hepatic encephalopathy; however, no patient discontinued the therapy. Although this study is small number and retrospective study, the results suggest that even if the ammonia level increases after initiation of lenvatinib therapy, therapy might be able to continue by the use of an appropriate intervention or combination of interventions, including withdrawal, dose reduction, medication, or shunt occlusion.

This study has limitations. First, the sample size of our study is very small unfortunately. However, there is no previous report about hyperammonemia or hepatic encephalopathy in patients treated with lenvatinib. In our knowledge, this is the first report about close observation of ammonia levels. Thus, we considered our report is valuable even if the sample size is very small. Second, we could not evaluate real-time changes in intrahepatic blood flow at baseline and after administration of lenvatinib.

In conclusion, in patients with advanced HCC who have PSCs treated by lenvatinib, we should be aware of the early manifestation of hyperammonemia because of their decreased liver function before initiation of treatment. However, we think that appropriate management might enable the continuation of lenvatinib therapy, even with the development of hyperammonemia during the therapy.
